# Simulation and analysis of non-navigational errors in robot-assisted pedicle Kirschner wire placement surgery

**DOI:** 10.1186/s13018-025-05790-4

**Published:** 2025-05-02

**Authors:** Yongkang Yang, Yishi Jia, Chang Liu, Liang Li, Boyao Wang

**Affiliations:** 1 Nanjing Medical University, Nanjing, 211166 China; 2 The Second Affiliated Hospital of Nanjing Medical University, Nanjing, 210003 China

**Keywords:** Non-navigational errors, Precision measurement, Orthopedic surgical robots, Surgical simulation, Pedicle Kirschner wire placement

## Abstract

**Background:**

Surgical errors of orthopedics robotic are influenced by a multitude of factors. This study aims to investigate the impact of non-navigational errors on the accuracy of pedicle screw placement in orthopedic surgery.

**Methods:**

Initially, a robot-assisted Kirschner wire (K-wire) placement simulation system was constructed, comprising a universal arm, wide-angle cameras, microscope cameras, and a vertebral base. Utilizing this system, we conducted a systematic analysis of the effects of four factors on non-navigational errors: operator habits, guide-to-bone surface distance, robotic arm stiffness, and vertebral fixation stiffness.We investigated two distinct operator habits: Habit 1 involves first positioning the K-wire against the bone surface through the guide and then inserting it using a bone drill; Habit 2 involves clamping the K-wire onto the bone drill and then inserting it together. Based on the control variable method, we designed precision measurement experiments for K-wire placement under different factors, forming 26 variable combinations to investigate the K-wire placement errors under each factor and their proportions in the overall error.

**Results:**

A total of 933 K-wire placements were performed in this study. The average deviation under Habit 2 conditions was 0.51 mm, compared to 0.13 mm under Habit 1 conditions; the average deviation was 0.36 mm when the guide-to-bone surface distance was 5 cm, and 0.28 mm when the distance was 1 cm; the average deviation was 0.36 mm under the 600 mm robotic arm condition, and 0.24 mm under the 500 mm robotic arm condition; the average deviation was 0.37 mm in the Plaster-Fixed Vertebra Group, and 0.85 mm in the Silicone-Fixed Vertebra Group.

**Conclusions:**

Operator habits and vertebral fixation stiffness are the primary factors influencing non-navigational errors, while guide-to-bone surface distance and robotic arm stiffness are secondary factors. This study recommends adopting Habit 1 in clinical surgeries, minimizing the guide-to-bone surface distance, and enhancing the stiffness of the robotic arm and vertebral fixation to reduce non-navigational errors and improve the accuracy of robot-assisted pedicle screw placement.

## Introduction

With the rapid development of minimally invasive surgical techniques and biomedical engineering, robot-assisted surgery has emerged as a pivotal tool in clinical applications [1–3]. These surgical procedures offer distinct advantages, including high precision, reduced radiation exposure, and shorter operative times [4, 5]. In the field of orthopedic surgery, robots have been widely utilized in various procedures, such as percutaneous vertebroplasty, percutaneous pedicle screw fixation, bone biopsy, and sacroiliac screw fixation [6–8]. For instance, the TINAVI surgical robot (TINAVI Medical Technologies Co., Ltd, Beijing, China) has been deployed in over 90,000 surgeries, demonstrating remarkable accuracy and reliability [9–11].

Surgical safety and patient prognosis are of common concern to both clinicians and patients [12]. In robot-assisted orthopedic surgery, the placement of the K-wire is a crucial step for guiding the surgical pathway, and its precision directly affects the therapeutic efficacy and patient prognosis.Depending on their operational mechanisms of current navigation-based surgical robots, errors in robot-assisted K-wire placement can be primarily categorized into two sources: navigational and non-navigational.

Navigational errors are those arising from registration, tracking, and movement of the robotic arm within the surgical robot system. These errors can be mitigated through optimization of robotic surgical navigation methods. Non-navigational errors, conversely, occur after the robotic arm has reached its designated position. These are caused by factors such as the operator habits, stiffness of the robotic arm, rigidity of vertebral fixation, and distance between the guide and the bone surface, which prevent the K-wire from following the pre-designed trajectory accurately. These errors are unrelated to surgical navigation methods.

Although there are numerous reports on the accuracy of orthopedic robotic surgery, the focus has overwhelmingly focused on navigational accuracy or overall precision [13–15]. Research concerning non-navigational errors remains underexplored. In particular, there is a significant gap in systematic studies addressing which factors influence the accuracy of K-wire placement and to what extent these factors impact the precision.

**Fig. 1:**
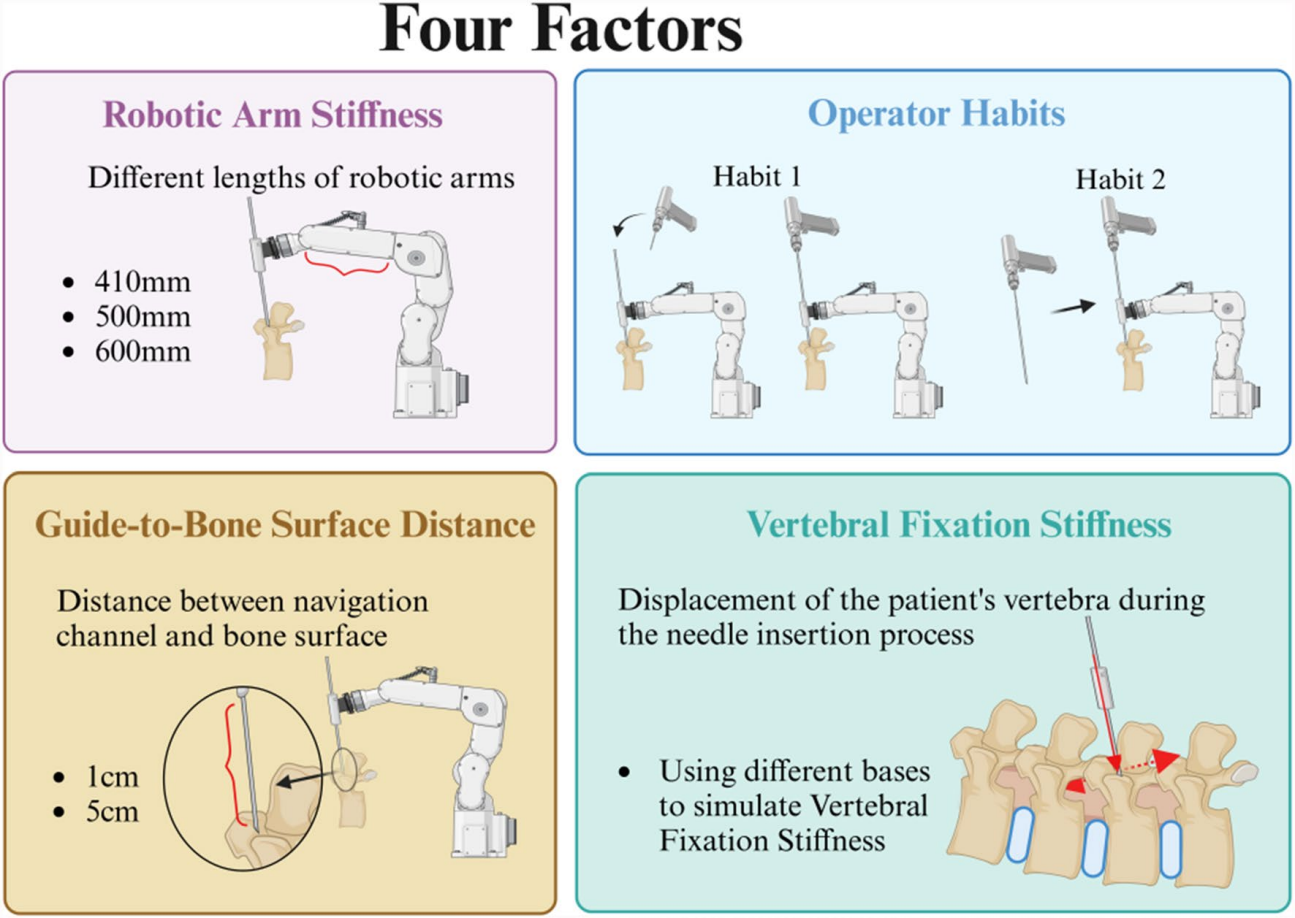
Four influencing factors on the precision of robot-assisted K-wire placement

Our clinical observations and system analysis reveal four critical sources of non-navigational errors (Fig. 1). The primary factor involves operator habits. Following robotic arm positioning, subsequent surgical procedures are executed by the surgeon. Variations in operator habits and the sequence of K-wire placement can affect the precision of K-wire placement. The second critical parameter is the guide-to-bone surface distance, defined as the distance from the end of the guide to the bone surface. During robot-assisted K-wire placement, the guide controls and constrains the direction of the K-wire. Once the K-wire exits the guide, it is no longer constrained and may deviate significantly from the planned trajectory. The third factor is the robotic arm stiffness. When subjected to external forces, the robotic arm may experience oscillations, which affects the precision of K-wire placement. The amplitude of oscillation is directly correlated with the stiffness of the robotic arm [16]. The final element is the vertebral fixation stiffness. The vertebrae, linked by muscles and ligaments, may not be sufficiently stable. During K-wire placement, they may shift due to external forces, thereby compromising the precision of K-wire placement.

To investigate the effects of Non-Navigational Errors on the precision of orthopedic robotic surgery, this study established a laboratory-based K-wire placement simulation system. This system was specifically designed to replicate the process of robot-assisted K-wire placement and to analyze the influencing factors and extent of non-navigational errors, thereby providing a reference for clinical practitioners.

## Methods

**Fig. 2:**
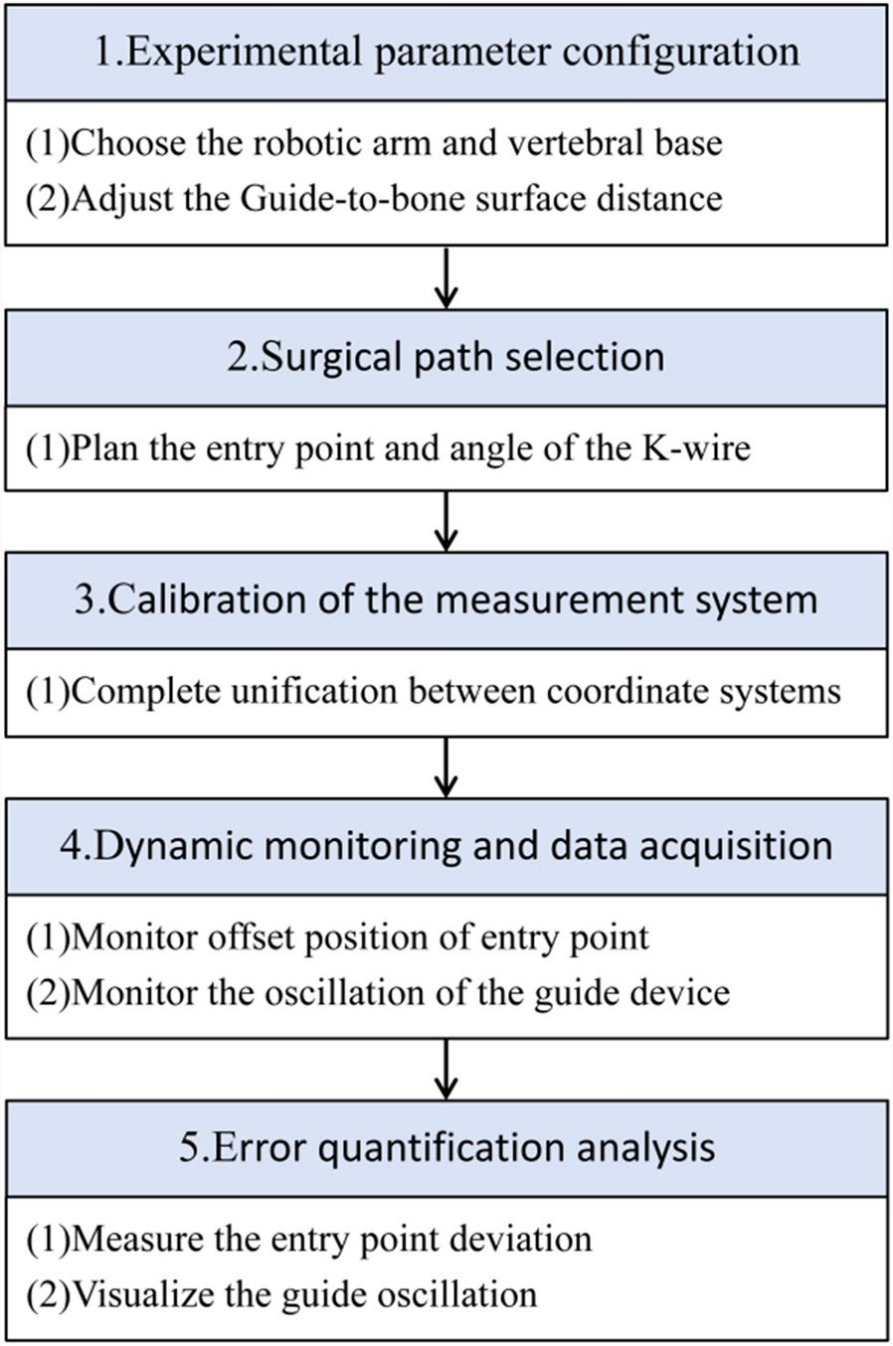
Workflow of the K-wire placement simulation system. Experimental parameter configuration refers to adjusting the simulation system setup according to the combinations of influencing factors to achieve different experimental conditions. Surgical path selection refers to planning the entry point and angle of the K-wire. Dynamic monitoring and data acquisition refer to the complete monitoring of the K-wire tip position and guide oscillation throughout the entire needle placement process. Error quantification analysis involves processing the data from the previous step to measure the entry point deviation and visualize the guide oscillation

This study investigates the impact of various factors on non-navigational errors using the Robot-Assisted K-Wire Placement Simulation System. The main steps involved in the system operation include: experimental parameter configuration, surgical path selection, calibration of the measurement system, dynamic monitoring and data acquisition, and error quantification analysis (Fig. 2).

### Construction of the robot-assisted K-wire placement simulation system

Given ethical restrictions and patient safety requirements, human/animal studies for non-navigational error analysis are impractical. Furthermore, regulatory constraints associated with surgical robot manufacturing significantly restrict access to robotic systems with adjustable parameters, including variable robotic arm stiffness levels. To address this limitation, this study constructed a robot-assisted K-wire placement simulation system under laboratory conditions. The experimental framework enables controllable simulation and analysis through three key aspects: system structural design, surgical path planning, and error measurement methodologies.

#### System components and workflow

**Fig. 3:**
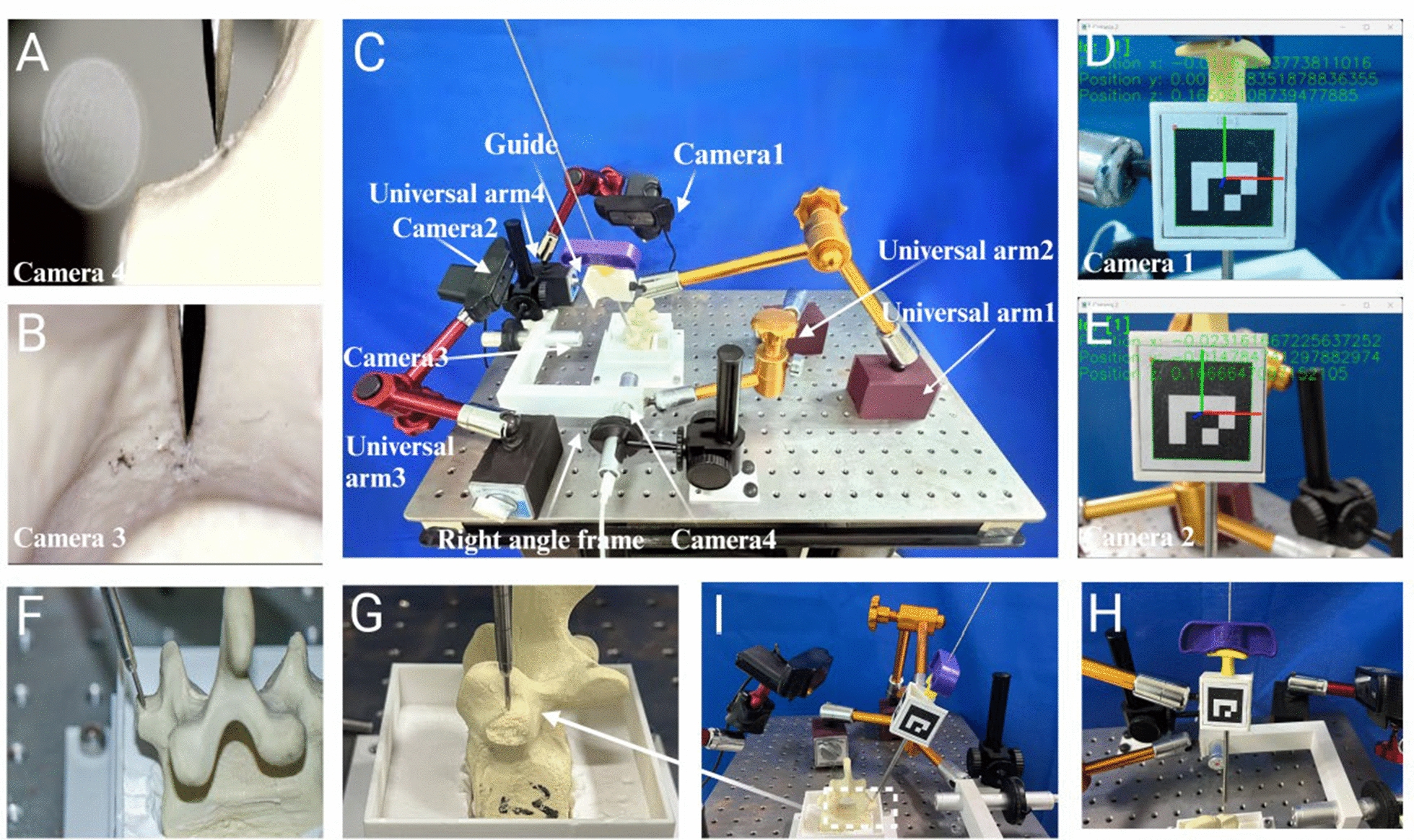
Components of the robot-assisted K-wire placement simulation system. **A** and **B** depict the images captured by Cameras 4 and 3, respectively. **C** presents an overall view of the simulation system, which includes three articulated arms for securing the K-wire placement pathway and cameras, four cameras in total (with the lower two mounted on a right-angle bracket), and a guide adorned with ArUco markers. **D** and **E** correspond to the images from Cameras 1 and 2, respectively. **F** and **G** show the K-wire in contact with the vertebrae from frontal and lateral perspectives, while **H** and **I** illustrate the ArUco markers on the guide as viewed from the frontal and lateral angles

The system comprises a universal arm, guide, cameras, a support frame, and vertebral components (Fig. 3). The universal arm functions to simulate the robotic arm of a surgical robot, enabling the adjustment and planning of K-wire placement position and angle. The system is equipped with four cameras to monitor the deviation of the entry point and the oscillation of the guide. Specifically, two wide-angle cameras (Camera1 and Camera2) record guide oscillations (Fig. 3D and E), while two microscopes (Camera3 and Camera4) track K-wire tip deviations (Fig. 3A and B). The system incorporates a navigation module designed with 3D printing technology to simulate the navigation device mounted on the robotic arm’s end-effector. The exterior of the navigation module is equipped with tracking QR codes (Fig. 3I and H) o track real-time positional and angular changes during K-wire placement. To maintain clinical procedural fidelity, the system integrates an orthopedic bone guide into the navigation module to form the guide, directing the insertion of the K-wire. The final K-wire placement procedure is completed using a hollow bone drill followed by K-wire insertion.

The operational workflow of the system is illustrated in Fig. 3. Initially, the combinations of influencing factors are identified. Subsequently, based on the selected influencing factors, choose the appropriate universal arm and set the guide-to-bone surface distance. Following this, adjust the position and angle of the guide are adjusted to define the K-wire placement position and angle. Upon completion of the K-wire placement, the cameras are utilized to record the entire process, and image and coordinate data are collected and documented. Finally, the obtained image and coordinate data are processed in the computer system, and the results are compared under different conditions.

#### Measurement of entry point deviation error

Two high-magnification microscope cameras are utilized to capture the tip of the K-wire from the frontal and lateral perspectives to record the deviation of the K-wire tip. Since the cameras cannot observe the actual position of the K-wire tip after it has entered the vertebra, this study adopts the positional deviation of the entry point as the measurement criterion. The entry point is defined as the contact location between the K-wire tip and osseous surface during insertion initiation (Fig. 9). During the K-wire insertion process, it is only necessary to press the K-wire tip against the bone surface, causing a slight puncture, without the need to insert the K-wire deeply into the vertebral body. The deviation can subsequently be measured using the microscope camera system.

***Calibration of the Measurement System Coordinate System:*** Before each K-wire placement and image acquisition, it is essential to calibrate the coordinate system of the measurement apparatus, as this serves as the basis for subsequent decomposition and measurement of entry point deviation [17]. In the dual-microscope entry point deviation measurement system developed for this study, there are three spatial Cartesian coordinate systems are incorporated: the frontal microscope camera coordinate system, the lateral microscope camera coordinate system, and the guide coordinate system.

The guide coordinate system constitutes the fundamental reference framework of this system. The coordinate system is defined with the tip of the K-wire as the origin, where the *Z*-axis extends upward along the direction of the K-wire, and the *X*–*Y* plane is perpendicular to the axis of the K-wire. In the frontal microscope camera coordinate system, the *Y*-axis extends in the direction of the camera’s field of view, whereas the *X*-axis and *Z*-axis correspond to the horizontal and vertical directions of the field of view, respectively. For the lateral microscope camera coordinate system, the *X*-axis extends in the direction of the camera’s field of view, while the *Y*-axis and *Z*-axis corresponding to the horizontal and vertical directions of the field of view, respectively.

**Fig. 4:**
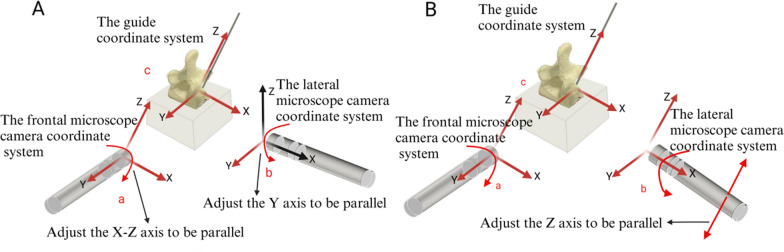
The method for calibrating the coordinate system of the measurement system is shown in the figure, which indicates the adjustment directions of the cameras and displays the coordinate systems after alignment in two different scenarios. **A** shows the three coordinate systems used in the experiment: a represents the frontal microscope camera coordinate system, b represents the lateral microscope camera coordinate system, and c represents the guide coordinate system. Red coordinate axes in the figure indicate that they have been aligned parallel to the guide coordinate system, while black coordinate axes indicate that they have not been aligned to parallel. **A** shows the coordinate system alignment process when the K-wire tip is not obstructed, while **B** shows the alignment process when the K-wire tip is obstructed

The calibration protocol initiates with the guide coordinate system being employed as a reference to adjust the frontal microscope camera such that its *Z*-axis aligns parallel with that of the guide coordinate system, and the *X*-axis and *Y*-axis of the frontal microscope camera coordinate system are utilized to ascertain the guide coordinate system. The lateral microscope camera is subsequently adjusted in accordance with the frontal microscope camera. When the K-wire tip is unobstructed within the field of view of the frontal microscope camera, the frontal camera measures the deviation components along the *X*-axis and *Z*-axis, while the lateral camera measures the deviation along the *Y*-axis. At this point, it is only necessary to ensure that the *Y*-axis of the lateral camera aligns with the guide coordinate system. Due to the spatial relationships between the coordinate axes, the *X*–*Z* plane of the guide coordinate system and the lateral microscope camera coordinate system will be parallel to each other. However, the *X*-axis and *Z-*axis of the two systems are not parallel to each other (Fig. 4A). If the K-wire tip is obstructed within the field of view of the frontal microscope camera, the frontal camera measures the deviation along the *X*-axis, and the lateral camera measures the deviations along both the *Y*-axis and *Z*-axis. In this case, the *X*-axis, *Y*-axis, and *Z*-axis of the lateral microscope camera coordinate system must be precisely adjusted to align with the guide coordinate system (Fig. 4B).

**Fig. 5:**
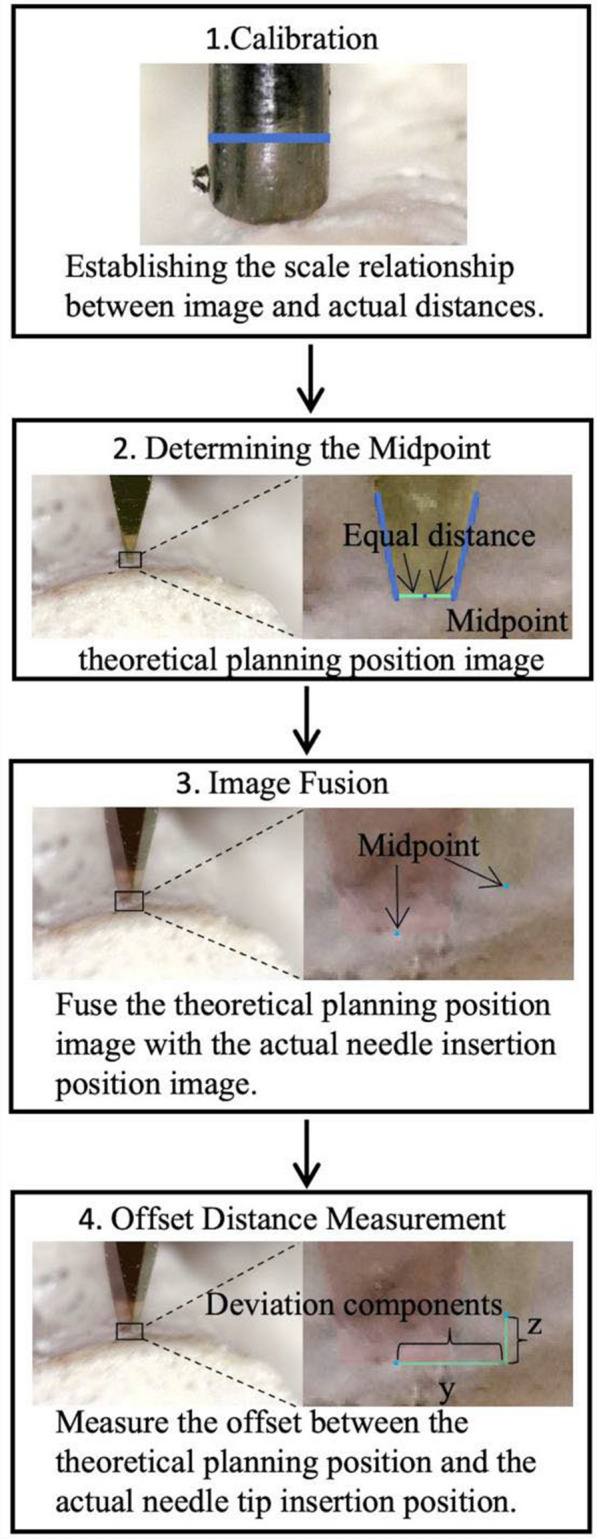
Steps for measuring entry point deviation. This figure illustrates the specific steps for measuring the deviation distance in the image captured by the lateral microscope camera. The steps for the frontal microscope camera are essentially the same. Step 2 shows how to determine the center point of the entry point in the theoretical image. The intersection point of the K-wire and the bone surface is found, and the center point is the point equidistant from both sides of the K-wire. The same method is used in the actual needle placement image. Step 4 shows the method for measuring the decomposed deviation distance in the lateral camera image. The horizontal deviation component is denoted as *y*, and the vertical deviation component is denoted as *z*. In the frontal camera image, the horizontal deviation component is denoted as x, and the vertical deviation component is denoted as *z*

***Camera Image Calibration:*** Owing to the presence of scale magnification in camera imaging, which depends on the distance between the camera and the K-wire, image calibration is required to establish the proportional relationship between lengths in the image and those in reality. The calibration process is illustrated in Fig. 5. In this study, the diameter of the K-wire is employed as a reference; its diameter is measured with a vernier caliper, and the range of pixels it occupies in the image is subsequently measured to complete the calibration process. It is critical to emphasize that calibration must be performed before each K-wire placement under different experimental conditions. This is attributed to the distance between the camera and the K-wire varies across different experimental groups, resulting in differences in magnification levels.

***Verification of Entry Point Deviation Error:*** Entry point deviation is resolved into three orthogonal directions: *X*, *Y*, and *Z*. Owing to the effects of perspective distortion, the images captured by the cameras cannot accurately measure the deviation along the depth of the field. Moreover, when the K-wire deviates in the direction of the camera’s field of view extension, it modifies the scaling of the image, thus compromising the calibrated proportionality and rendering inaccurate the distance measurements in the other two directions [18].

Assuming that the planned position of the K-wire is at a distance *U*_*1*_ from the microscope camera, and after deviation, the distance from the microscope camera is *U*_*2*_, the diameter of the K-wire is *D*_*1*_, the range of pixels occupied by the K-wire in the microscope camera image is *N*_1_, and the total range of pixels in the image is *N*_*2*_. The length of the image in the camera’s field of view at the planned position is *L*_*1*_, and at the deviated position is *L*_2_. Before and after the K-wire placement, the range of pixels occupied by the deviation component in one direction is *N*_3_. Based on preliminary experiments, under the laboratory conditions, *U*_*1*_ is approximately 10 cm, the deviation of the entry point is sub-millimeter, *N*_*2*_ is about 1150 pixels, *D*_*1*_ is 1.54 mm, and *N*_*1*_ is about 100 pixels. Therefore, we select *U*_*1*_ = 100 mm, *U*_*2*_ = 101 mm, *D*_*1*_ = 1.54 mm, *N*_*1*_ = 100, *N*_*2*_ = 1150, *N*_*3*_ = 50, to calculate the error *E* caused by the scale magnification after deviation:

$${L}_{1}=\frac{{N}_{2}{D}_{1}}{{N}_{1}}$$

$${L}_{2}=\frac{{L}_{1}{U}_{2}}{{U}_{1}}$$

$$E=\frac{{N}_{3}{L}_{3}}{{N}_{2}}-\frac{{N}_{3}{D}_{1}}{{N}_{1}}$$

By substituting the values into the calculation, we obtain *E*≈0.0077 mm, which is negligible under the precision conditions of this experiment.

***Acquisition of Deviation Before and After K-wire placement:*** Identify the midpoint of the entry point within the image. When the tip of the K-wire is clearly visible, the midpoint at the bone surface junction is designated as the entry point center (Fig. 5). When the K-wire tip is not clearly visible, the intersection point is identified by extending the edge line of the K-wire. However, this intersection point represents only the actual position of the K-wire tip, not the midpoint of the entry point, and therefore cannot be used to measure the deviation along the *Z*-axis. The theoretically planned position image was superimposed with the actual K-wire placement to measure the deviations along the *X*, *Y*, and *Z* axes, followed by calculation of the total deviation distance.

#### Measurement of guide oscillation error

The system utilizes two wide-angle cameras to monitor the oscillation of the guide from the frontal and lateral perspectives during the K-wire placement. The monitoring setup consisted of ArUco markers, cameras, and a computer system. ArUco, an OpenCV-based library, is specifically designed for pose estimation using marker codes [19, 20]. Identification markers are attached to both the front and side of the guide to determine the correspondence and spatial relationships among various parts of the guide [21]. Through observation and identification of the center point position, coupled with coordinate transformation and data logging in the computer system, the *X* and *Y* coordinates are recorded to accurately reflect the guide’s three-dimensional oscillation. Recording initiates before each K-wire placement and terminates after completion to comprehensively document the oscillation during the entire procedure.

The two wide-angle cameras can recognize the marker codes and generating the real-time *X*, *Y*, and *Z* coordinates of the center points of these markers. However, owing to the effects of perspective distortion, the cameras’ precision in detecting coordinates along the depth direction (*Z*-axis) is limited. Therefore, each camera captures only the *X* and *Y* coordinates. Through analyzing the motion of the guide in both the frontal and lateral planes, the overall oscillation of the guide in three-dimensional oscillation can be fully characterized.

To quantify the amplitude of oscillation of the guide, a Cartesian coordinate system were established for both the frontal and lateral views, using the midpoint coordinates of the guide’s markers prior to each K-wire placement serving as the origin. The real-time coordinate changes of the guide’s midpoint throughout the K-wire insertion process are recorded, and the coordinate data from the same experimental conditions are combined into the same coordinate system. A 95% confidence ellipse is then generated based on the coordinate data, and the area of the ellipse is employed to quantify the amplitude of oscillation.

### Experimental protocol design

#### Sample preparation

**Fig. 6:**
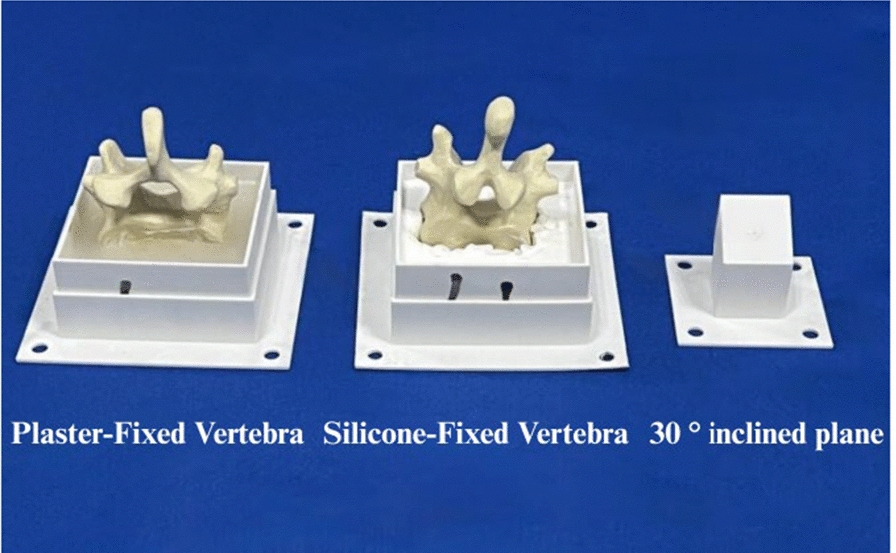
Fabrication of Various Specimens

A human lumbar vertebra simulation model (ENOVO, Shanghai, China) was employed (Fig. 6). This model represents a 1:1 replica in both size and shape, with the transverse processes’ outer edges truncated to enable lateral observation and measurement. A 30° inclined plane and a horizontal plane were fabricated using 3D printing technology to replicate the conditions for oblique K-wire placement on the bone surface and to perform system performance testing. Vertebrae L1 through L5 were positioned in 3D-printed boxes, and a plaster base was prepared by mixing plaster powder with water at a 3:1 ratio to prevent vertebral movement during K-wire placement. Furthermore, a silicone base was made using a 5° silicone mixture (Formulation A: Formulation B = 1:1) to replicate vertebral displacement during K-wire insertion.

#### Selection of surgical path

**Fig. 7:**
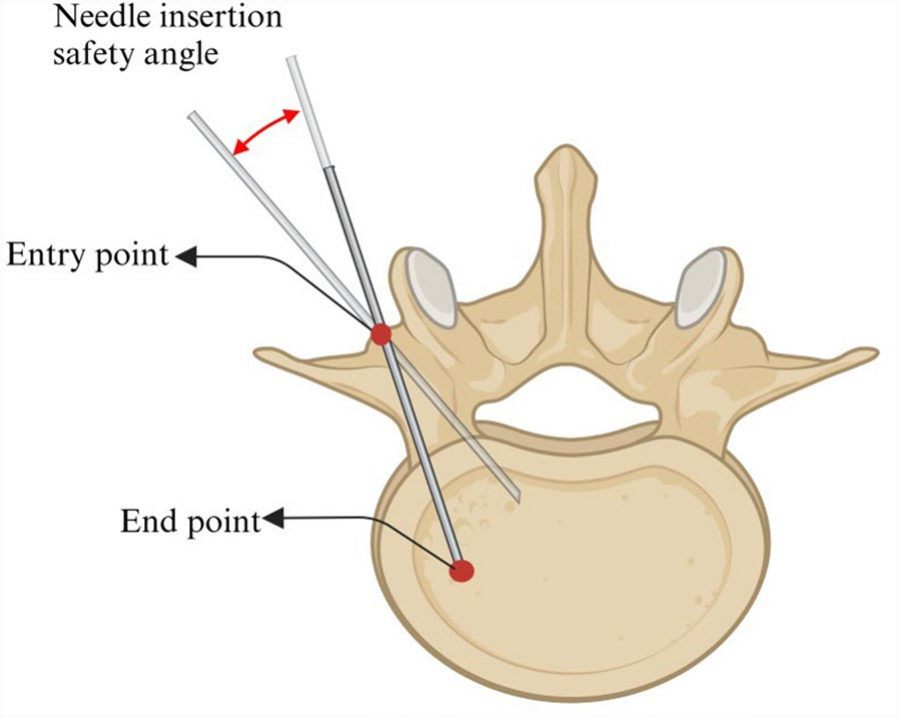
Entry point, End point, planning of entry points and placement angles

The planning the entry point and angle is a critical aspect of the experiment, since its accuracy directly influences surgical outcome and safety. In the Vertebral Group, the entry point was determined at the superolateral aspect of the vertebral arch, corresponding to the 10 o’clock position and the right vertebra at the 2 o’clock position for the right vertebra. This location minimizes the risk of medial wall puncture while improving surgical safety [22, 23]. K-wire placement at the physiological angle of the vertebral arch (Fig. 7) decreases the likelihood of medial wall puncture [24–26]. In the Inclined Plane Group, vertical K-wire placement was adopted, where the K-wire forming a 30° angle with the inclined plane, and the entry point was determined at the center of the inclined plane.

#### Determination and combination of experimental variables

**Fig. 8:**
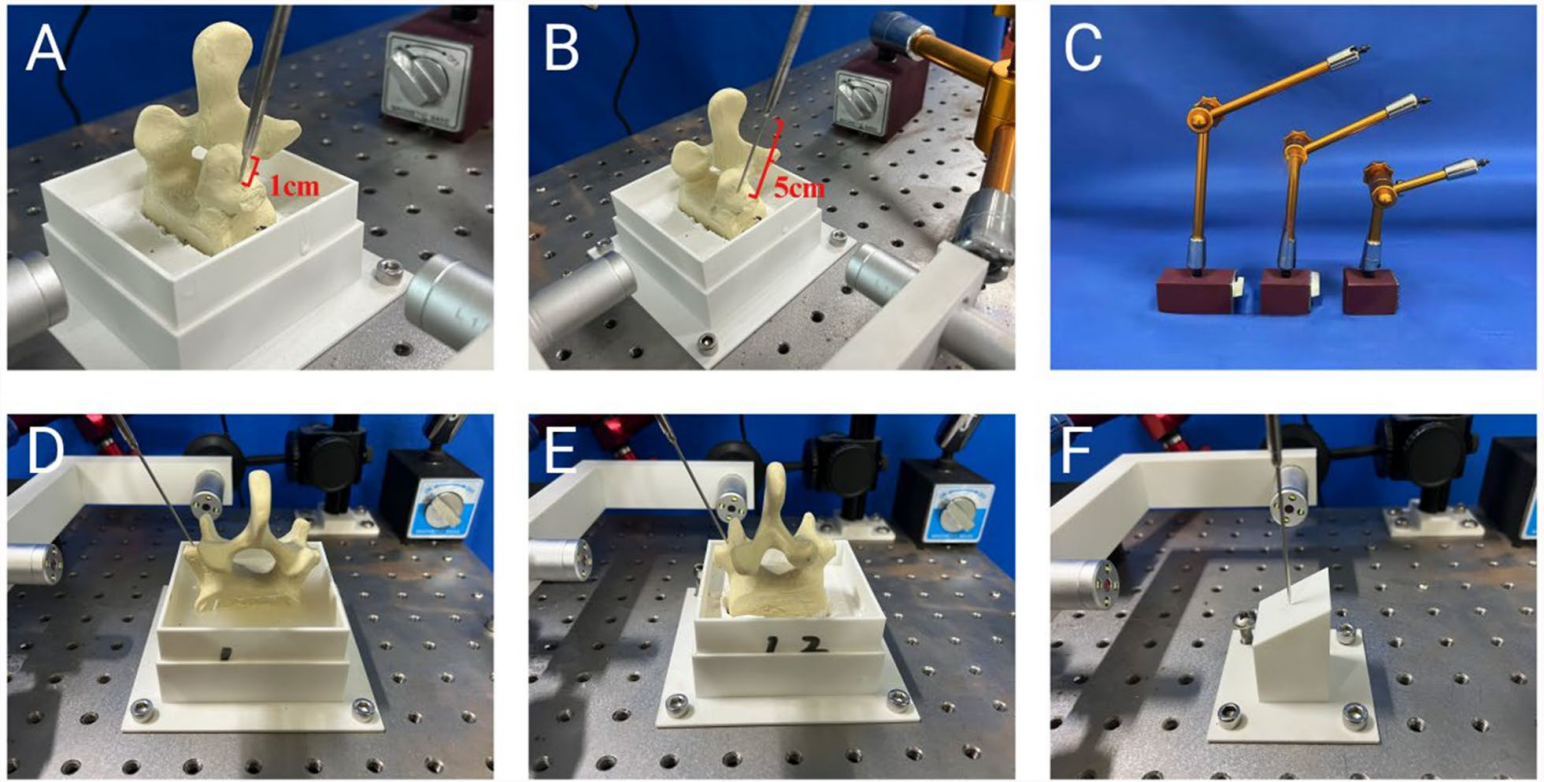
Illustration of Different Experimental Variable Combinations. Figs. **A** and **B** represent the influencing factor of the guide-to-bone surface distance, with A being 1 cm and **B** being 5 cm. Fig. **C** depicts mechanical arms of different lengths, specifically a 600 mm, 500 mm, and 410 mm arm. Figs. **D**, **E**, and **F** represent influencing factor related to vertebral fixation stiffness, which are the silicone base, plaster base, and the 30° inclined plane, respectively

This study investigated the effects of four influencing factors on the accuracy of K-wire placement. Regarding operator habits, two different habits were selected. The first habit (Habit 1) involves positioning the K-wire against the bone surface through the guide and then inserting it using a hollow bone drill. The second habit (Habit 2) involves clamping the K-wire in the bone drill and then inserting both together into the guide for needle placement. Regarding the guide-to-bone surface distance, distances of 1 cm and 5 cm were selected (Figs. 8A and B). Regarding robotic arm stiffness, mechanical arms with lengths of 410 mm, 500 mm, and 600 mm were selected to simulate different stiffness levels (Fig. 8C). Regarding vertebral fixation stiffness, plaster and silicone were used to fabricate vertebral bases to simulate two different fixation stiffness levels (Figs. 8D and E). Additionally, a 30° inclined plane was introduced for experimentation (Fig. 8F).

**Fig. 9:**
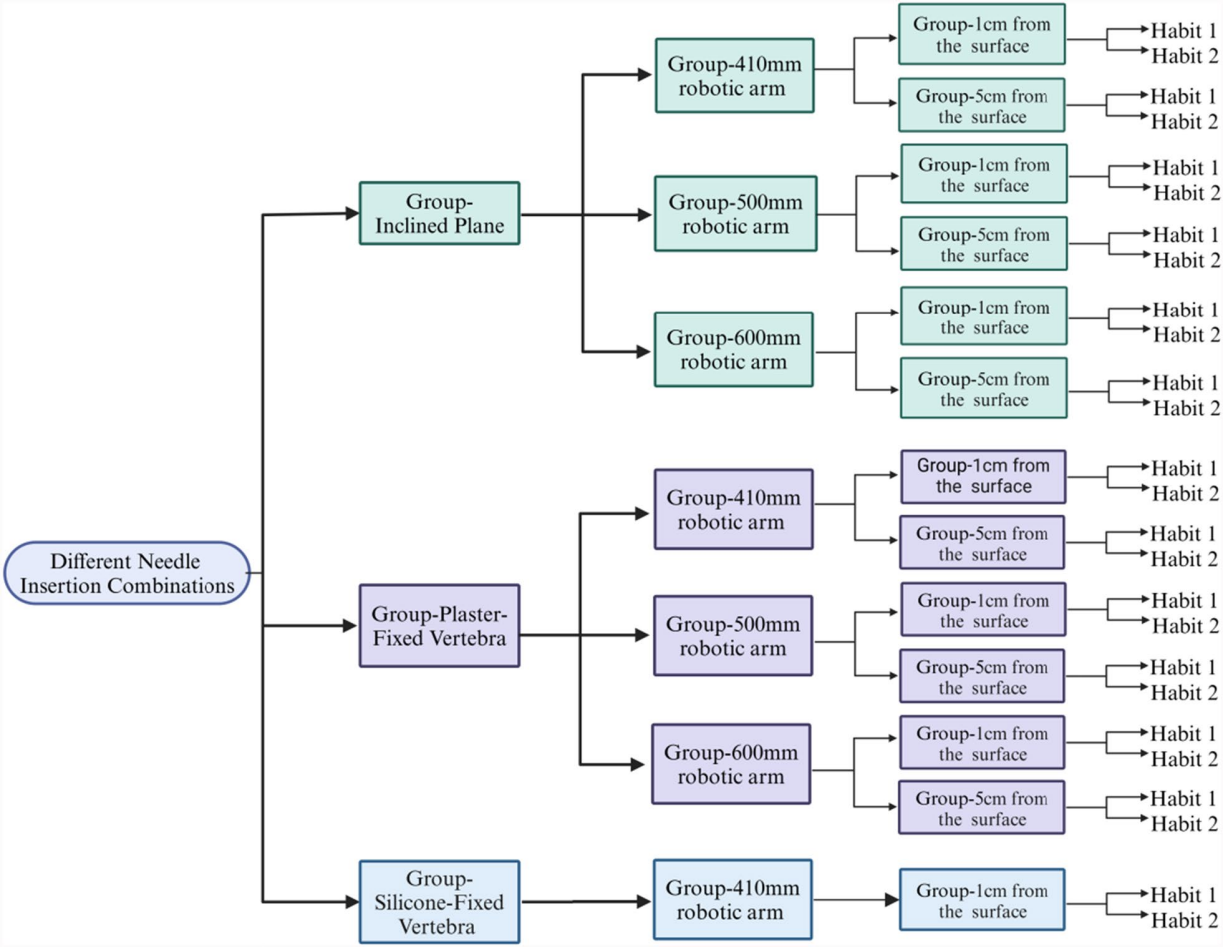
Twenty-six K-wire placement scenario combinations designed according to the principle of controlled variables

The previously mentioned influencing factors and conditions were systematically combined to create 26 experimental combinations (Fig. 9). The experimental groupings were organized as follows: in the Inclined Plane Group, K-wire insertion was performed with the plane aligned towards both the frontal and lateral microscope cameras. In the Vertebral Group, L1-L5 vertebra models were utilized, with K-wire placement executed on both left and right sides. To reduce interference from experimental variables, after completing each set of experiments, the operator habits were modified after each experimental set, while maintaining consistency in other conditions (such as K-wire placement position and angle). This approach enables for an accurate evaluation of the impact of operator habits on the precision of K-wire placement.

#### Selection of specific tools and measurement software

The bone guide employed for vertebral shaping was acquired from KaiLiTai Medical Device Co., Ltd (Shanghai, China). The hollow bone drills were procured from BoJin Medical Device Co., Ltd (Shanghai, China). The K-wires, measuring 1.5 mm in diameter and 40 mm in length, were supplied by Tianjin XinZhong Medical Device Co., Ltd (Tianjin, China). Wide-angle cameras were provided by Shanghai Tux Information Technology Co., Ltd (Shanghai, China), whereas the microscope cameras were procured from Shenzhen Chaoyan Technology Co., Ltd (Shenzhen, China). The articulated arms were produced by Dongguan TaiXin Hardware Tools Co., Ltd (Dongguan City, China).

The K-wire placement process was captured in real-time using OBS Studio version 27.2.4 (Open Broadcaster Software, USA). Image analysis and distance measurements were performed with Adobe Photoshop CC 2019 (Adobe, USA), which provides pixel-level precision measurement and image blending functionalities. Custom were developed in Python 3.6 (Python Software Foundation, Wilmington, NC, USA), utilizing the OpenCV 3.1.4.15 computer vision library for real-time ArUco marker recognition and camera pose synchronization. Statistical data analysis and graph plotting were conducted using Origin 2024 (OriginLab, Northampton, USA), guaranteeing high precision and reproducibility in result visualization.

## Results

### Performance testing of the K-wire placement simulation system

Considering that the positional discrepancy between the planned entry point and the actual entry point may stem from the deviation between the theoretical planned position and the actual placement of the K-wire (i.e., systematic error), or from the influencing factors under investigation. To quantify the systematic error, twenty theoretical entry point measurements were performed on both the horizontal plane and the 30° inclined plane at guide-to-plane distances of 1 cm and 5 cm. For each measurement, the K-wire was extracted from and reinserted in the guide to isolate systematic error as the sole source of deviation between planned and actual placement points. (It should be noted that Habit 1 involves placing the K-wire only once during planning and insertion, whereas Habit 2 requires the K-wire to be placed twice, thus this systematic error only affects Habit 2.)

A coordinate system was defined, using the first measurement point under each set of conditions as the origin. Subsequent measurement points were logged, and their coordinates were computed relative to the central point to derive the average distance, representing the range of systematic error fluctuation. As shown in Table 1, systematic error escalates with greater guide-to-bone surface distance (5 cm > 1 cm), and deviation values are elevated on the inclined plane compared to the horizontal plane. However, the total deviation attributable to systematic factors remains within 0.35 mm.

**Table 1:** Impact of systematic error on entry point deviation

	Mean value (mm)	Standard deviation (mm)
1 cm Horizontal Plane	0.13	0.07
5 cm Horizontal Plane	0.29	0.12
1 cm Inclined Plane	0.19	0.09
5 cm Inclined Plane	0.34	0.13

### Analysis of individual influencing factors

Following the establishment of the experimental variable combinations, we conducted simulated K-wire placement experiments were executed under various combination conditions. In the Inclined Plane Group, twenty K-wire placements were carried out for each combination (10 times with the plane facing the frontal camera and 10 times with it facing the lateral camera), resulting in 240 placements. In the Plaster-Fixed Vertebra Group, fifty placements were performed for each combination (5 on each side of each vertebra), yielding 600 placements. In the Silicone-Fixed Vertebra Group, fifty placements were executed for each combination (5 on each side of each vertebra), resulting in 100 placements. Following the exclusion of incomplete or corrupted image data, a total of 933 K-wire placements were analyzed.

#### Impact of operator habits on non-navigational errors

**Fig. 10:**
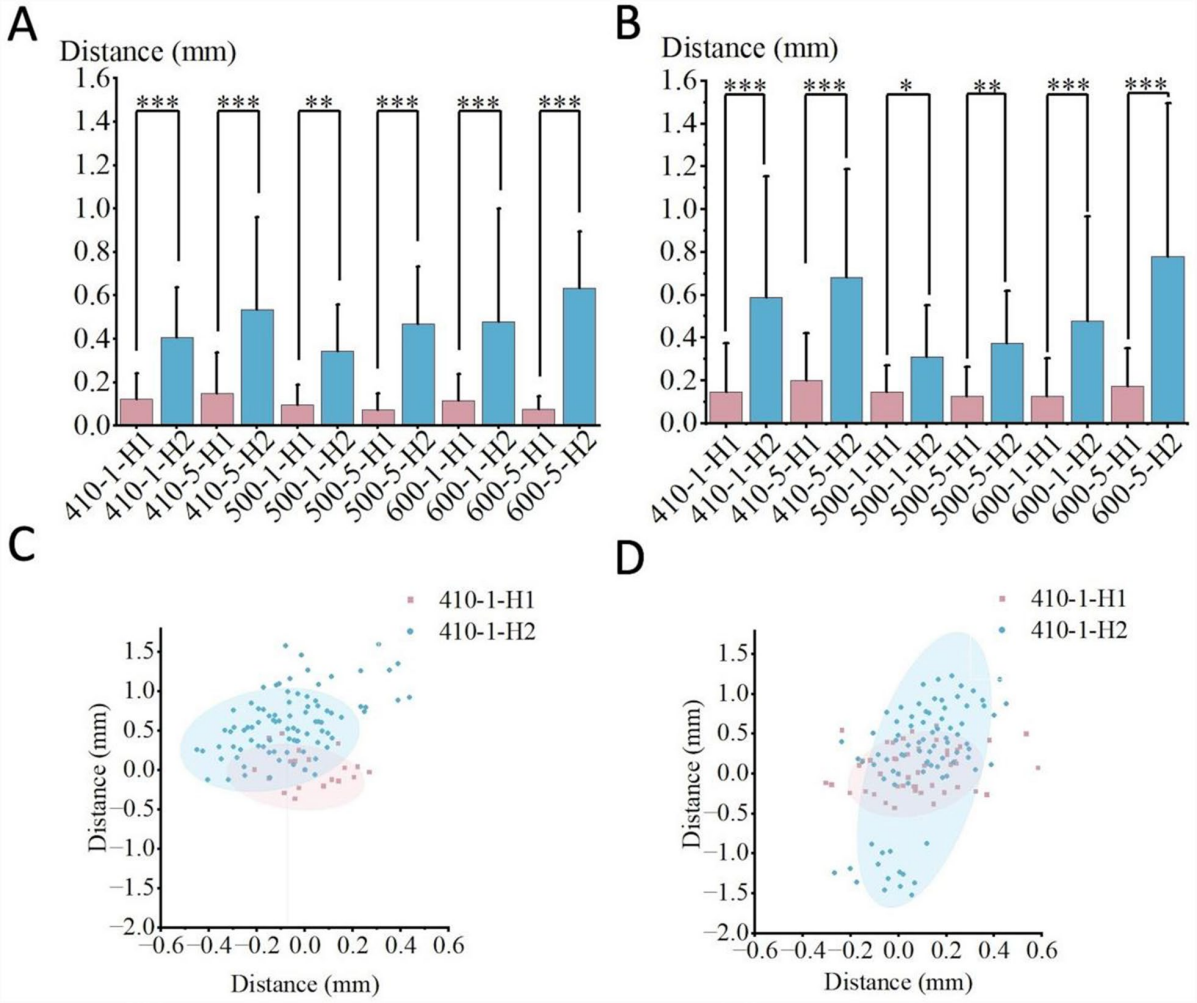
Statistical charts and scatter plots for the two operator habits show that Habit 2 has greater deviation distances and guide oscillation compared to Habit 1.Fig. **A** corresponds to the Inclined Plane Group, Fig. **B** to the Plaster-Fixed Vertebra Group, Fig. C to the frontal view, and Fig. D to the lateral view. The ellipses in Figs. **C** and **D** encompass 95% of the coordinates. In the present Figs. and subsequent images, H1/H2 denote Habit 1/Habit 2, respectively. The numbers 410/500/600 represent robotic arm lengths of 410 mm, 500 mm, and 600 mm, respectively. The numbers 1/5 denote guide-to-bone surface distances of 1 cm and 5 cm, respectively. Ca/Si denote the Plaster-Fixed Vertebra Group/Silicone-Fixed Vertebra Group. In the significance analysis, n.s. indicates P > 0.05, * indicates P ≤ 0.05, ** indicates P ≤ 0.01, and *** indicates P ≤ 0.001

The impact of operator habits on non-navigational errors is illustrated in Tables 2 and 3, and we utilized paired t-tests and Cohen’s d analysis to analyze the data [27, 28]. Meanwhile, Fig. 10 displays statistical charts of entry point deviation and scatter plots of guide oscillation for the two different habits.Among various combinations, Habit 2 demonstrated significantly greater entry point deviation and guide oscillation than Habit 1.

**Table 2:** Deviation under various habit combinations

	Plaster-Fixed Vertebra Group							Inclined Plane Group					
	Habit 1		Habit 2			T		Habit 1		Habit 2		T	
	$\overline{\text{x} }$	σ	$\overline{\text{x} }$	σ		P	d	$\overline{\text{x} }$	σ	$\overline{\text{x} }$	σ	P	d
410–1	0.15	0.23	0.59		0.57	***	1.95	0.12	0.12	0.49	0.23	***	2.02
410–5	0.20	0.22	0.68		0.50	***	1.24	0.15	0.19	0.53	0.43	***	1.14
500–1	0.15	0.12	0.31		0.24	*	0.84	0.10	0.09	0.34	0.22	**	1.43
500–5	0.13	0.14	0.37		0.24	**	1.22	0.08	0.08	0.47	0.26	***	2.03
600–1	0.13	0.18	0.48		0.49	***	0.95	0.12	0.12	0.48	0.52	***	0.95
600–5	0.18	0.18	0.78		0.71	***	1.16	0.08	0.06	0.64	0.26	***	2.97

In this table and subsequent tables, H1 and H2 denote Habit 1 and Habit 2, respectively. The numbers 410, 500, and 600 refer to robotic arm lengths of 410 mm, 500 mm, and 600 mm, respectively. The numbers 1 and 5 indicate guide-to-bone surface distances of 1 cm and 5 cm, respectively. Ca and Si represent the Plaster-Fixed Vertebra Group and Silicone-Fixed Vertebra Group, respectively. The symbol $\overline{x }$ denotes the mean value in millimeters (mm), σ denotes the standard deviation in millimeters (mm) T denotes the Paired T-test and d denotes the Cohen’s d. n.s. indicates P > 0.05, * indicates P ≤ 0.05, ** indicates P ≤ 0.01, and *** indicates P ≤ 0.001.We used paired t-tests to compare the statistical significance of differences between conditions and additionally employed Cohen’s d analysis to quantify the effect sizes and associations under different conditions [27, 28]

**Table 3:** Area Covered by 95% confidence ellipses under different condition combinations

	Area (mm^2^)		
	Frontal View	Lateral View	Total
410–1-H1-Ca	0.75	0.46	1.21
410–1-H2-Ca	0.37	1.31	1.68
410–5-H2-Ca	0.95	0.98	1.93
500–1-H2-Ca	1.09	1.31	2.40
600–1-H2-Ca	2.17	1.98	4.15
410–1-H2-Si	3.99	1.88	5.87

#### Impact of guide-to-bone surface distance on non-navigational errors

**Fig. 11:**
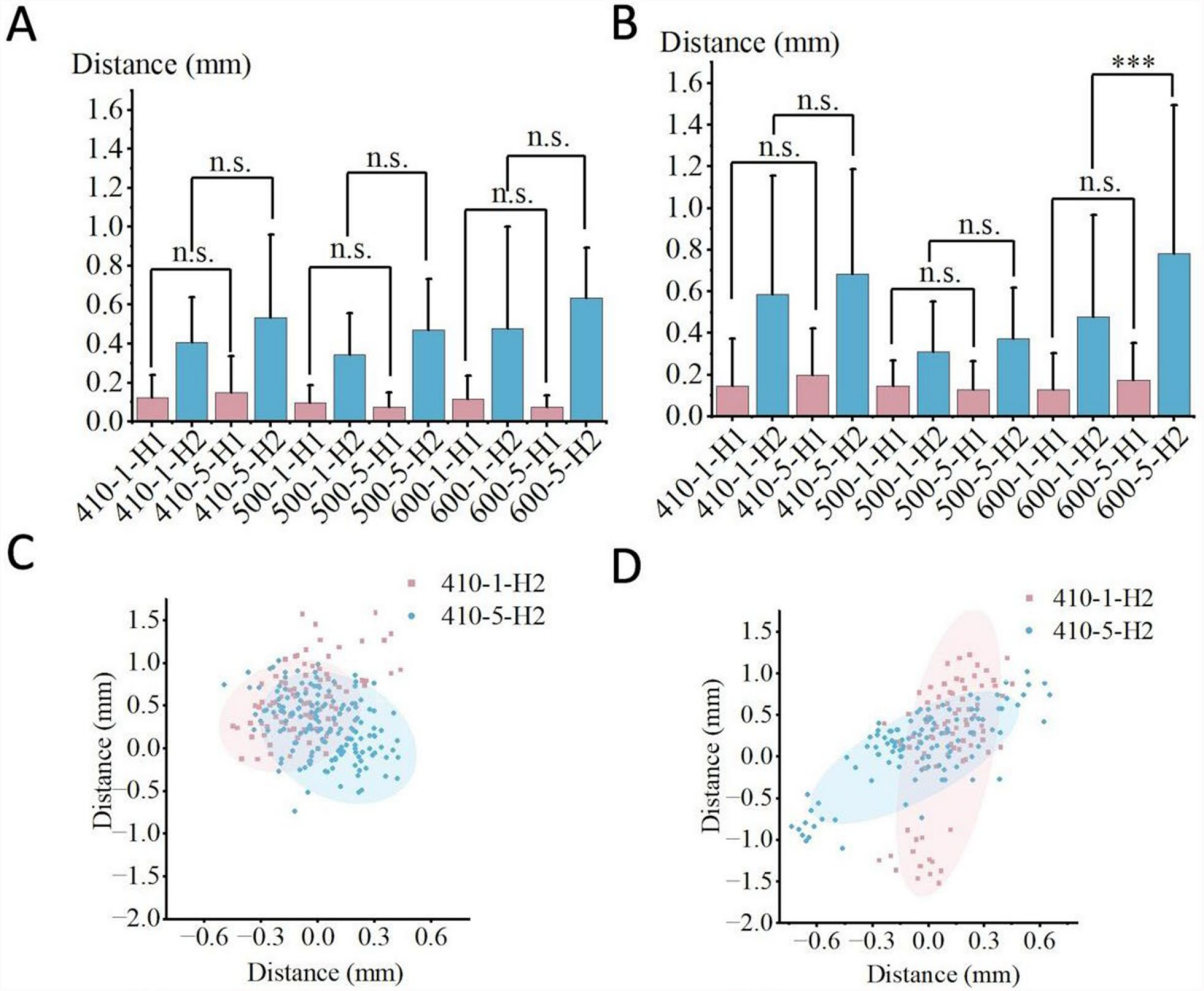
Statistical charts and scatter plots for the two guide-to-bone surface distance conditions show that, under Habit 2, the deviation and guide oscillation are greater when the guide-to-bone surface distance is 5 cm compared to 1 cm.Fig. **A** represents the Inclined Plane Group, Fig. **B** represents the Plaster-Fixed Vertebra Group, Fig. **C** represents the frontal view, and Fig. **D** represents the lateral view

The effect of guide-to-bone surface distance on non-navigational errors is presented in Tables 3 and 4, while Fig. 11 presents the statistical charts of entry point deviation and the scatter plots of guide oscillation for the two distances. In Habit 1 conditions, the effect of different distances on the magnitude of deviation was not significant. However, under Habit 2 conditions, the deviation and the oscillation amplitude of the navigation channel were both significantly higher at a distance of 5 cm than at 1 cm.

**Table 4:** Data for each group condition at different guide-to-bone surface distances

	Plaster-fixed vertebra group						Inclined plane group					
	1 cm		5 cm		T		1 cm		5 cm		T	
	$\overline{\text{x} }$	σ	$\overline{\text{x} }$	σ	P	d	$\overline{\text{x} }$	σ	$\overline{\text{x} }$	σ	P	d
410-H1	0.15	0.23	0.20	0.22	n.s	0.22	0.12	0.13	0.15	0.19	n.s	0.18
410-H2	0.59	0.57	0.68	0.50	n.s	0.17	0.41	0.23	0.53	0.43	n.s	0.35
500-H1	0.15	0.12	0.13	0.14	n.s	0.15	0.10	0.09	0.08	0.08	n.s	0.23
500-H2	0.31	0.24	0.37	0.24	n.s	0.25	0.34	0.22	0.47	0.26	n.s	0.54
600-H1	0.13	0.18	0.18	0.18	n.s	0.28	0.12	0.12	0.08	0.06	n.s	0.42
600-H2	0.48	0.49	0.78	0.71	***	0.50	0.48	0.52	0.64	0.26	n.s	0.39

#### Impact of robotic arm stiffness on non-navigational errors

**Fig. 12:**
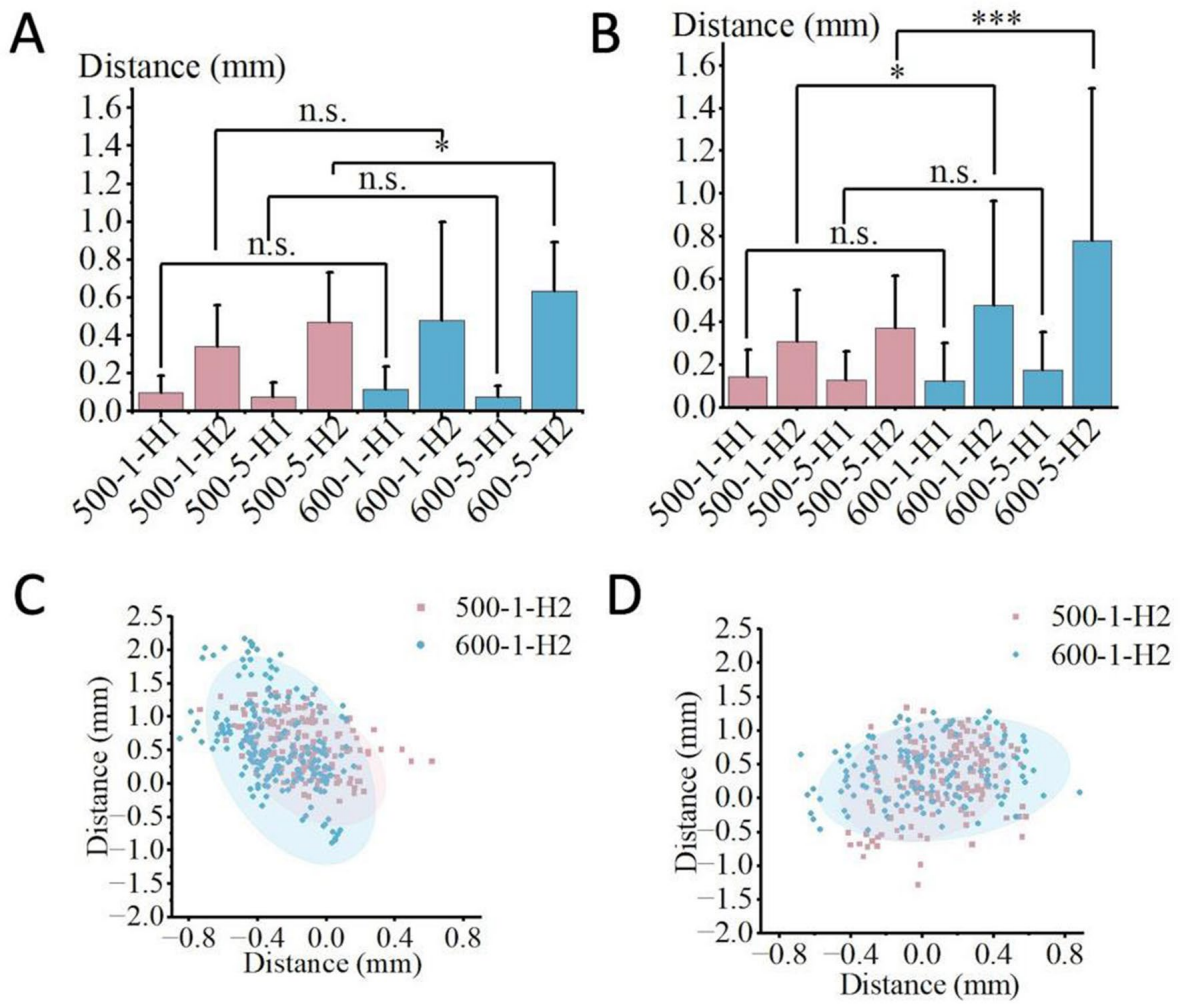
Statistical charts and scatter plots for the two robotic arm stiffness conditions show that, under Habit 2, the deviation and guide oscillation are greater with the 600 mm arm compared to the 500 mm arm.Fig. **A** corresponds to the Inclined Plane Group, Fig. **B** to the Plaster-Fixed Vertebra Group, Fig. **C** to the frontal view, and Fig. **D** to the lateral view

The effect of robotic arm stiffness on non-navigational errors is depicted in Tables 2, 3, and 4, while Fig. 12 displays statistical charts of entry point deviation and scatter plots of guide oscillation for two robotic arm lengths. In Habit 1 conditions, the difference in entry point deviation between the two arm lengths was not significant. However, under Habit 2 conditions, the deviation and oscillation of the navigation channel were significantly greater for the 600 mm arm than for the 500 mm arm across various combinations.

#### Impact of vertebral fixation stiffness on non-navigational errors

**Fig. 13:**
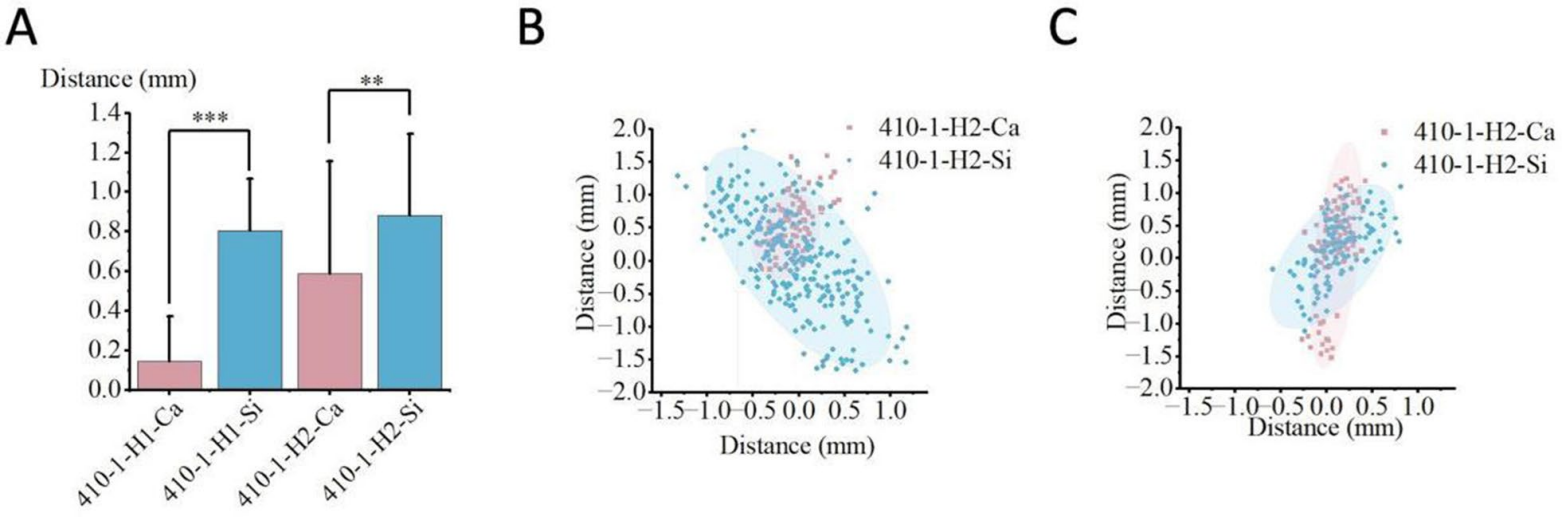
Statistical charts and scatter plots for the two vertebral fixation stiffness conditions show that the deviation and guide oscillation are significantly greater in the Silicone-Fixed Vertebra Group compared to the Plaster-Fixed Vertebra Group. Fig. **A** represents the comparison of the deviation between the Plaster-Fixed Vertebra Group and the Silicone-Fixed Vertebra Group. Fig. **B** represents the frontal view, and Fig. **C** represents the lateral view

The effect of operator habits on non-navigational errors is shown in Tables 3 and 5, while Fig. 13 presents statistical charts of entry point deviation and scatter plots of guide oscillation for two vertebral fixation stiffness conditions. Among various combinations, the Silicone-Fixed Vertebra Group demonstrated significantly greater entry point deviation and guide oscillation than the Plaster-Fixed Vertebra Group.

**Table 5:** Data on deviation under different vertebral fixation stiffness conditions

	Plaster-fixed vertebra group		Silicone-fixed vertebra group		T	d
	$\overline{\text{x} }$	σ	$\overline{\text{x} }$	σ		
410–1-H1	0.15	0.23	0.81	0.26	***	2.68
410–1-H2	0.59	0.57	0.88	0.41	**	0.58

### Proportional analysis of error influencing factors

**Fig. 14:**
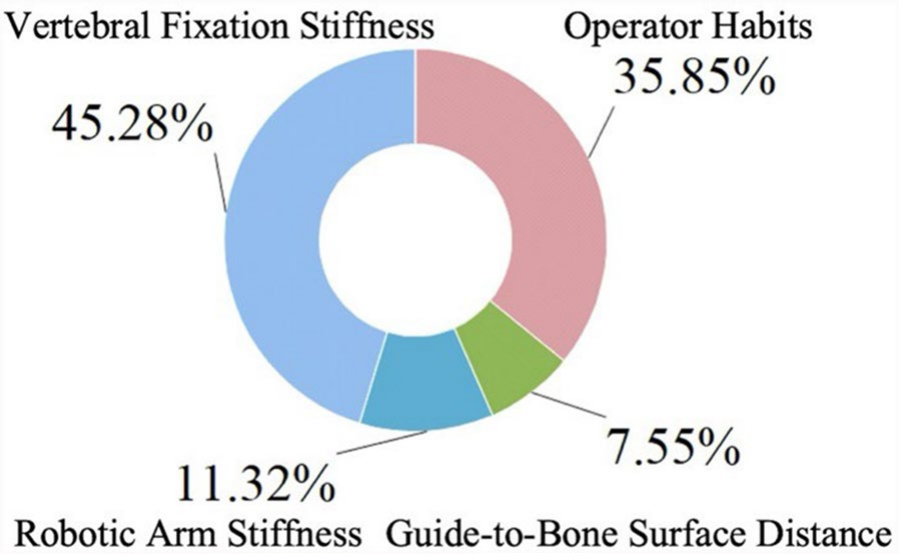
Analysis of the Proportions of Four Influencing Factors

A thorough analysis of the effects of the aforementioned four factors on non-navigational errors is provided in Table 6 and Fig. 14. Under the experimental conditions of this study, vertebral displacement caused the greatest deviation, followed by operator habits, while the guide-to-bone surface distance and robotic arm stiffness resulted in smaller deviations. Thus, it can be concluded that operator habits and vertebral fixation stiffness are the primary factors, whereas guide-to-bone surface distance and robotic arm stiffness are secondary factors.

**Table 6:** Analysis of the proportional impact of error influencing factors

	Operator habits	Guide-to-bone surface distance	Robotic arm stiffness	Vertebral fixation stiffness
Deviation(mm)	0.38	0.08	0.12	0.48

### Proportional analysis of error influencing factors

A thorough analysis of the effects of the aforementioned four factors on non-navigational errors is provided in Table 6 and Fig. 14. Under the experimental conditions of this study, vertebral displacement caused the greatest deviation, followed by operator habits, while the guide-to-bone surface distance and robotic arm stiffness resulted in smaller deviations. Thus, it can be concluded that operator habits and vertebral fixation stiffness are the primary factors, whereas guide-to-bone surface distance and robotic arm stiffness are secondary factors.

## Discussion

Investigating the factors influencing the precision of surgical robots is of significant importance for guiding clinical applications. Among these, the factors influencing non-navigational errors constitute an area requiring further exploration. However, direct investigation of these factors faces significant limitations in clinical settings, including ethical considerations, patient safety concerns, and the complexity of practical procedures. This study successfully addressed these constraints by developing a robot-assisted K-wire placement simulation system, which provides a robust experimental platform for systematically exploring the factors that influence non-navigational errors.

Concerning operator habits, K-wire insertion under Habit 2 conditions led to greater deviation and guide oscillation, demonstrating that the operator habits significantly influence the precision of K-wire placement. The effect of Habit 1 was less pronounced, likely because the weight of the K-wire itself, which causes the tip to slightly penetrate the bone surface, reducing its susceptibility to deviation. This also explains why other influencing factors are not as evident under Habit 1 conditions during K-wire placement. Thus, it is recommended to adopt Habit 1 for K-wire placement in surgical procedures.

Concerning the vertebral fixation stiffness, the deviations under various conditions in the Silicone-Fixed Vertebra Group were significantly greater than those in the Plaster-Fixed Vertebra Group, and a similar trend was observed for guide oscillation. This indicates that vertebral displacement during K-wire placement is an important factor influencing the precision of K-wire placement, and under the conditions of this simulation experiment, its effect is the most significant. Although the vertebral fixation method in the simulation does not perfectly replicate the actual fixation in the human body, it sufficiently demonstrates that vertebral displacement during K-wire placement significantly influences the accuracy of K-wire placement.

**Fig. 15:**
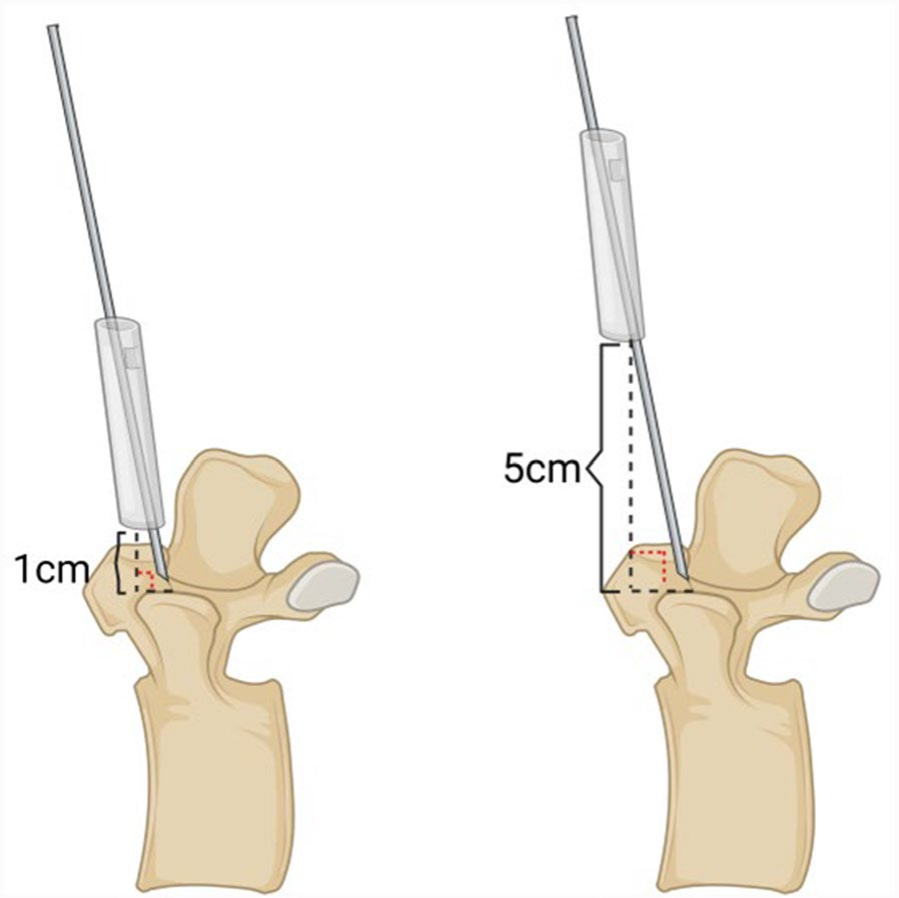
The maximum displacement caused by varying distances from the bone surface

Concerning the guide-to-bone surface distance, a K-wire with a diameter of 1.54 mm is inserted through a guide with an inner diameter of 2 mm. This difference in diameter permits a certain degree of wobble of the K-wire within the channel, which is likely the primary cause of the observed errors. Calculations show that at a distance of 1 cm from the bone surface, the maximum displacement is 0.26 mm, whereas at a distance of 5 cm, the maximum displacement can reach 0.38 mm (Fig. 15). During K-wire insertion under Habit 2 conditions, the displacement at 5 cm is greater than that at 1 cm, and the wobble within the navigational channel aligns with these findings. Thus, in clinical applications, it is recommended positioning the guide as close to the bone surface as possible.

Concerning the stiffness of the robotic arm, a comparison was conducted between a 500 mm robotic arm and a 600 mm robotic arm. The sole difference between these two arms is their length, with all other parameters identical, making stiffness the only variable. The results indicated that the displacement caused by the 500 mm arm was lower than that caused by the 600 mm arm. Similarly, the guide wobble was also reduced with the 500 mm arm. This demonstrates that robotic arm stiffness significantly influences the precision of K-wire placement, with increased stiffness enhancing accuracy. Thus, it is recommended that the stiffness of the robotic arm be appropriately increased during surgical robot production to improve precision.

Regarding the two influencing factors—guide-to-bone surface distance and robotic arm stiffness—no significant differences in displacement were observed under Habit 1 conditions. In contrast, under Habit 2 conditions, some groups did not exhibit statistically significant differences. The absence of significant differences in K-wire placement under Habit 1 conditions may be attributed to the initial placement of the K-wire tip into the bone surface during placement through the guide. This likely establishes a more stable starting point, thereby minimizing the influence of other factors on K-wire displacement. Under Habit 2 conditions, all groups showed greater displacement and guide wobble at a guide-to-bone surface distance of 5 cm compared to 1 cm, and with a robotic arm length of 600 mm compared to 500 mm. These findings highlight the impact of guide-to-bone surface distance and robotic arm stiffness on the accuracy of K-wire placement. The absence of statistical significance may result from the insufficient gradient differences set in the experimental design. In future experiments, we intend to increase the gradient differences to further validate these findings.

Although the non-navigational error factors examined in this study were derived from laboratory simulations, they have a close relevance to clinical practice. First, regarding the influence of operator habits, different perators exhibit distinct habits after the robot completes its guidance, and the forces applied during needle insertion also vary, which is reflected in the two needle insertion habits examined in this study.Second, when navigational errors are minimized, the distance between the robot guide and the bone surface can be adjusted. Our experiments demonstrate that reducing this distance leads to a decrease in non-navigational error. Regarding the robotic arm stiffness factor, the stiffness of robotic arms varies among manufacturers and also depends on the surgical path. For example, the stiffness decreases as the arm extends further for longer paths, whereas it is relatively better for shorter paths. Additionally, the patient’s vertebrae may shift due to external forces during needle insertion, and factors such as muscle and fat content can influence the degree of displacement. Our experiments comparing plaster and silicone vertebrae further verified this conclusion.

**Fig. 16:**
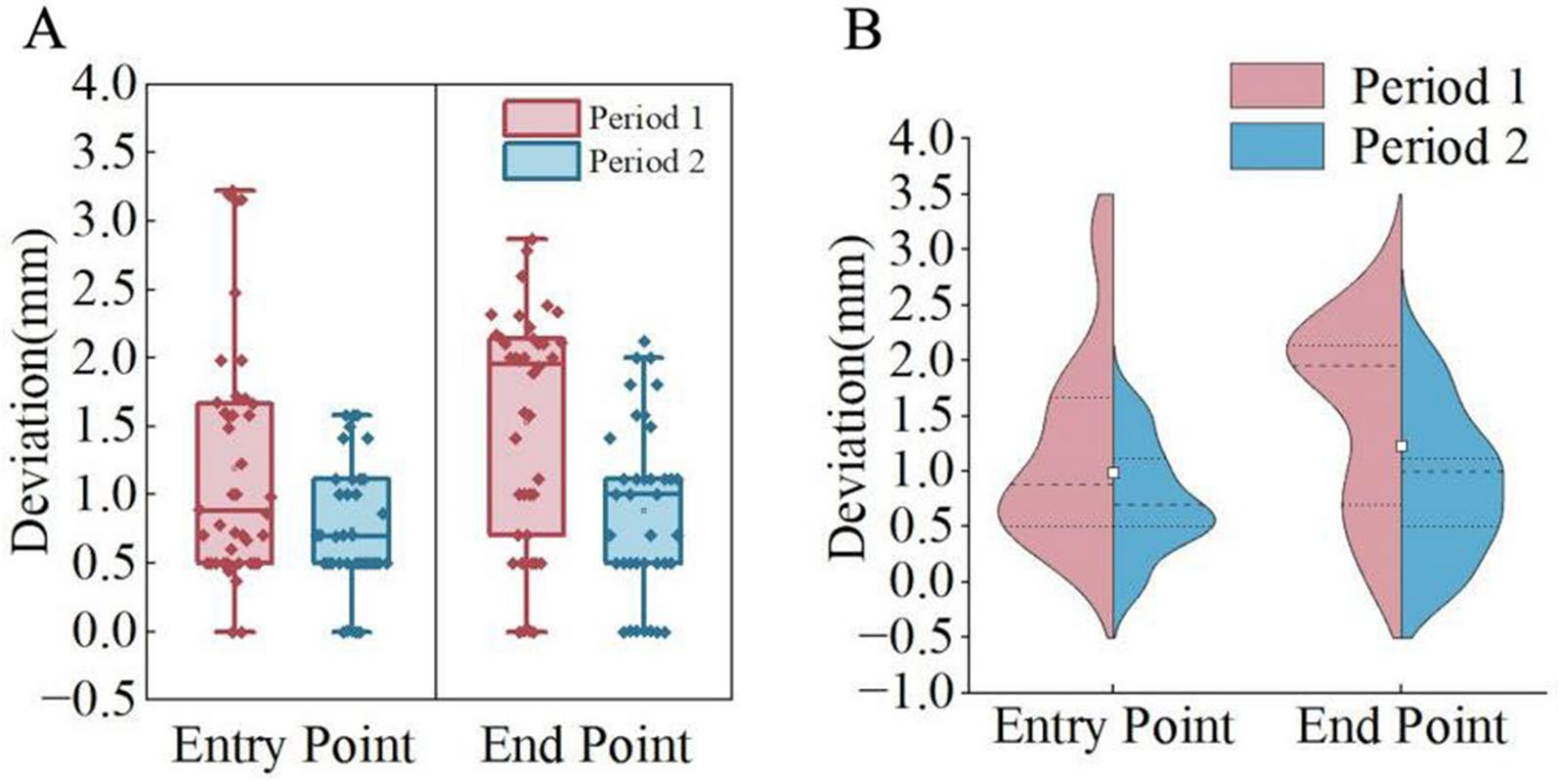
Box-and-whisker plots and violin plots for Period 1 and Period 2 show that the entry and endpoint deviations in Period 1 are significantly greater than those in Period 2

Our clinical data further support the findings of this study. We retrospectively reviewed the data of 27 patients who underwent robot-assisted pedicle screw placement surgery at the Second Affiliated Hospital of Nanjing Medical University (Nanjing, Jiangsu Province, China) from 2022 to 2024. The orthopedic robotic system used was the PL300B model (Nanjing Perlove Medical Equipment Co., Ltd, China), which has been certified for accuracy and performance by the National Medical Products Administration of China (NMPA). All surgeries were performed by the same surgical team using the same robotic system. In the initial phase of the surgeries, the operators followed the manufacturer’s instructions and used Habit 2 for needle insertion after the robotic arm reached its position (this phase is referred to as Period 1). After recognizing that needle insertion habits might affect surgical accuracy, subsequent patients underwent needle insertion using Habit 1 (this phase is referred to as Period 2). Period 1 included 14 patients with 42 needle insertions, exhibiting an entry point deviation of 1.19 ± 0.87 mm and an endpoint deviation of 1.53 ± 0.84 mm. Period 2 included 13 patients with 37 needle insertions, exhibiting an entry point deviation of 0.74 ± 0.47 mm and an endpoint deviation of 0.89 ± 0.63 mm. We validated these differences using paired t-tests and effect size analysis, with results shown in Table 7, revealing statistically significant differences in errors between the two phases. The data in Fig. 16 indicate that the surgeon’s operative habits, which we investigated, have a clinically significant impact on non-navigational errors. Due to ethical and technical limitations, we were unable to find clinical data on the impact of different guide-to-bone surface distances, robotic arm stiffness, and vertebral fixation stiffness on surgical accuracy. However, we believe that the simulation experiments conducted in this study largely demonstrate the impact of these non-navigational errors on surgical accuracy. From the perspective of the authors as surgical practitioners, these studies highlight the importance of considering non-navigational error factors in robot-assisted surgery, which is highly meaningful for our peers.

**Table 7:** Analysis of K-wire placement

	Period 1	Period 2	P	d
Number of patients	14	13		
Needle placements	42	37		
Average entry point deviation(mm)	1.19	0.74	< 0.01	0.63
Average end point deviation(mm)	1.53	0.89	< 0.005	0.85

## Conclusion

Our simulation studies on K-wire placement indicate that operator habits and vertebral fixation stiffness significantly affect the accuracy of robot-assisted K-wire placement. Furthermore, the guide-to-bone surface distance and robotic arm stiffness are recognized as important factors influencing procedural accuracy. Thus, it is recommended using Habit 1 for K-wire insertion, positioning the guide as close to the bone surface as possible, and increase the stiffness of both the robotic arm and vertebral fixation. The robot-assisted K-wire placement simulation system developed in this study is highly valuable for investigating factors contributing to non-navigational errors.

## Data Availability

No datasets were generated or analysed during the current study.

## Abbreviations

K-wire: Kirschner wire
H1: Habit 1
H2: Habit 2
410: Robotic arm lengths of 410 mm
500: Robotic arm lengths of 500 mm
600: Robotic arm lengths of 600 mm
1: Guide-to-bone surface distances of 1 cm
5: Guide-to-bone surface distances of 1 cm
Ca: Plaster-fixed vertebra group
Si: Silicone-fixed vertebra group
n.s.: P > 0.05
*: P ≤ 0.05
**: P ≤ 0.01
***: P ≤ 0.001
$$\overline{\text{x} }$$: Mean value
σ: Standard deviation
T: Paired T-test
d: Cohen’s d
mm: Millimeter
Fig: Figure
